# Serine Protease(s) Secreted by the Nematode *Trichuris muris* Degrade the Mucus Barrier

**DOI:** 10.1371/journal.pntd.0001856

**Published:** 2012-10-11

**Authors:** Sumaira Z. Hasnain, Michael A. McGuckin, Richard K. Grencis, David J. Thornton

**Affiliations:** 1 Immunity, Infection and Inflammation, Mater Medical Research Institute, Brisbane, Australia; 2 Manchester Immunology Group, Faculty of Life Sciences, University of Manchester, Manchester, United Kingdom; 3 Wellcome Trust Centre for Cell-Matrix Research, Faculty of Life Sciences, University of Manchester, Manchester, United Kingdom; University of Edinburgh, United Kingdom

## Abstract

The polymeric mucin component of the intestinal mucus barrier changes during nematode infection to provide not only physical protection but also to directly affect pathogenic nematodes and aid expulsion. Despite this, the direct interaction of the nematodes with the mucins and the mucus barrier has not previously been addressed. We used the well-established *Trichuris muris* nematode model to investigate the effect on mucins of the complex mixture of immunogenic proteins secreted by the nematode called excretory/secretory products (ESPs). Different regimes of *T. muris* infection were used to simulate chronic (low dose) or acute (high dose) infection. Mucus/mucins isolated from mice and from the human intestinal cell line, LS174T, were treated with ESPs. We demonstrate that serine protease(s) secreted by the nematode have the ability to change the properties of the mucus barrier, making it more porous by degrading the mucin component of the mucus gel. Specifically, the serine protease(s) acted on the N-terminal polymerising domain of the major intestinal mucin Muc2, resulting in depolymerisation of Muc2 polymers. Importantly, the respiratory/gastric mucin Muc5ac, which is induced in the intestine and is critical for worm expulsion, was protected from the depolymerising effect exerted by ESPs. Furthermore, serine protease inhibitors (Serpins) which may protect the mucins, in particular Muc2, from depolymerisation, were highly expressed in mice resistant to chronic infection. Thus, we demonstrate that nematodes secrete serine protease(s) to degrade mucins within the mucus barrier, which may modify the niche of the parasite to prevent clearance from the host or facilitate efficient mating and egg laying from the posterior end of the parasite that is in intimate contact with the mucus barrier. However, during a T_H_2-mediated worm expulsion response, serpins, Muc5ac and increased levels of Muc2 protect the barrier from degradation by the nematode secreted protease(s).

## Author Summary

Gastrointestinal parasitic worm infections cause significant morbidity, affecting up to a third of the world's populationand their domestic pets and livestock. Mucus, the gel-like material that blankets the surface of the intestine, forms a protective barrier that is an important part of our innate immune system. The whipworm *Trichuris* is closely associated with the intestinal mucus barrier. The major structural component of this barrier, large glycoproteins known as mucins play a significant role in the expulsion of these worms in a mouse model. Using mice that get longterm chronic infections and others able to expel the worms from the intestine, we uncover a novel role for products secreted by the worms. Enzymes secreted by whipworms can disrupt the mucin network that gives mucus its viscous properties. Moreover, we unravel that worm products are unable to degrade forms of mucins present in the mucus barrier during worm expulsion, suggesting that these enzymes may be released by the worm as part of its regime to improve its niche and survival in the host. However, the host is capable of producing mucins and other protective molecules that protect the mucus barrier from degradation and are detrimental to the viability of the worm.

## Introduction

Immune mediated elimination of gastrointestinal (GI) parasitic nematodes has been a subject of considerable investigation [1]. Hyperplasia of goblet cells that produce the secreted mucosal barrier is one of the most prominent features of the T_H_2-type immune response necessary for the expulsion of these pathogens from the intestine [1], [2]. However, until recently, definition of the precise role of goblet cells in host protection remained elusive, especially with regards to the major secreted component of goblet cells, the mucins, which are pivotal to the formation of the mucus layer that overlies the intestinal epithelium. Using established gastrointestinal nematode models *Trichuris muris*, *Trichinella spiralis* and *Nippostrongylus brasiliensis*, we have recently demonstrated that mucins are critical in resolving infection [3], [4], [5]. The major intestinal mucin Muc2 plays a significant role in the concerted protective worm expulsion mechanism and in its absence *T. muris* expulsion is significantly delayed [4]. Additionally, the Muc5ac mucin, not usually expressed in the murine intestine but induced post-infection during a T_H_2-type immune response, was demonstrated to be necessary for intestinal worm clearance [3]. Furthermore, Muc5ac was shown to directly affect the viability of the nematode. Bearing in mind that under field conditions GI nematodes can survive for long periods of time, it raises the question of how these parasites interact within the mucosal barrier and subvert the responses against them.

It is well established that GI nematodes secrete a variety of molecules (Excretory Secretory Products, ESPs) into the surrounding niche. These can be highly immunogenic, although, their functions *in vivo* are not well described [6]. *T. muris* infection in the mouse provides a unique tractable model that can be used to examine the interaction of parasites with the mucosal barrier during both acute (worm clearance by T_H_2 immune response) and chronic infection (lack of worm clearance by T_H_1 immune response) [6]. ESPs are thought to be very well-conserved and similar in terms of their antigenicity in the different *Trichuris* species. A study by Drake and co-workers has shown that a major 43 kDa protein secreted by the *Trichuris* nematode has the ability to induce ion-conducting pores in a lipid bilayer [7], whereas other studies have attributed the tunnel formation through the intestinal epithelium to protease activity (zinc metalloproteases, thiol protease and phenol oxidase) present in ESPs [8], [9].

In this study, we aimed to investigate the effect of the *T.muris* ESPs on the mucus barrier and in particular, on mucins which are an essential part of the co-ordinated T_H_2-mediated worm expulsion response. Our findings demonstrate for the first time that serine protease(s) secreted by the nematode have the ability to degrade Muc2 and depolymerise the mucin network. In contrast, the mucus barrier formed during worm expulsion is protected from ESP degradation. Particularly, Muc5ac mucin, which is necessary for worm expulsion, was resistant to ESP protease activity. Interestingly, in the mice that expel the nematodes, up-regulation of host serine protease inhibitors (Serpins) is observed which probably provides an additional level of protection of mucins, particularly Muc2, from degradation.

These data, therefore, demonstrate that GI nematode parasites produce protease(s) that degrade the major structural scaffold of the mucus barrier during chronic infection, resulting in a more porous mucus barrier, which in turn can aid establishment and persistence of nematode infection and/or exacerbate inflammation.

## Materials and Methods

### Animals

BALB/c (Harlan Olac) mice were maintained in the Biological Services Unit at The University of Manchester in a conventional clean *Helicobacter hepaticus-* and *norovirus-*free facility. All mice (6–12 wk old) were kept in sterilized, filter-topped cages, and fed autoclaved food in the SPF facility.

### Ethics statement

The protocols were employed at the University of Manchester and were performed in accordance with guidelines set and approved by the Animal Procedures Committee and Home Office Scientific Procedures Act (1986), United Kingdom under the personal licence issued to SZH (No. PIL: 40/9777).

### Parasitological technique

The techniques used for *T. muris* maintenance and infection were described previously [10]. Mice were orally infected with approximately 150 eggs for a high dose infection and <15 eggs for a low dose infection. Worm burdens were assessed by counting the number of worms present in the caecum. Worms present in the caecum on day 12 confirmed that infection had established in both groups of mice (high dose and low dose infection). Adult worms present in the caecum of the low dose infected group of mice, on day 35 post infection, confirmed chronic infection (Figure S1). ESPs were isolated *ex vivo* from adult *T. muris* nematodes using the method previously described [11]. ESPs were separated into <5 kDa, 5–50 kDa, 50–100 kDa and >100 kDa fractions using size exclusion Amicon columns (Millipore Pty Ltd, Australia).

### LS174T cell culture

Human intestinal adenocarcinoma LS174T cells (European Collection of Cell Culture, Salisbury, U.K) were used as a source of glycosylated MUC2. Cells were cultured with complete medium containing DMEM, 2 mM L-glutamine, 100 U/ml penicillin, 100 µg/ml streptomycin and 10% heat inactivated FBS (all from Invitrogen, Paisley, U.K) at 37°C in a humidified incubator (95% air with 5% CO_2_). When confluent, cells were gently scraped until dislodged from the flasks surface and rinsed with approximately 5 ml of complete media. The harvested cells were dispersed using a 25G needle and resuspended at approximately 1×10^6^ cells/ml [12].

### Expression of N-terminal Muc2

MKN45 gastric cancer cells that express mucin-specific chaperones but no endogenous MUC2 were transfected with the murine Muc2 N-terminal D3 domain (rMuc2-D3) consisting of 3 FLAG tags at the N-terminus and a myc tag at the C-terminus as previously described [13]; rMuc2-D3 was obtained from cell lysates using TRIS-HCl and Triton-X lysis buffer. Conditioned media was collected from transfected MKN45 cells and concentrated to obtain secreted rMuc2-D3.

### Antibodies

Immunodetection was carried out using a polyclonal antibody raised against a murine Muc2 (mMuc2) [4] or human MUC2 (hMUC2) [14]. Commercially available 45M1 antibody was used for the detection of mouse Muc5ac [3] and, mouse monoclonal FLAG antibody-clone 2 (Sigma-Aldrich, UK) and c-Myc antibody (9E10) was used to detect rMuc2-D3 [13].

### Murine mucus isolation

To isolate the mucus from mice, the caecum was gently flushed with PBS to remove the faecal matter, subsequently scraped lightly with a cell scraper into equal volumes of PBS and stored at −80°C until required.

### Cell culture mucus isolation

To isolate the mucus from LS174T cells, media was removed and cells were washed vigorously with ice cold PBS, mucus was stored at −80°C until required.

### ESP treatment

Mucus, mucins and rMuc2-D3 were incubated at 37°C with the ESPs for various time points (as specified) at final concentration of 50 µg/ml or above. Control samples were not treated with the ESPs, but were incubated at 37°C for the maximum time point. ESPs were heat inactivated at 100°C before incubation or incubated at 4°C with mucus as negative controls. ESP activity was quenched using the protease inhibitors: ethylenediaminetetraacetic acid (EDTA), N-ethylmaleimidide (NEM), Lupeptin, Chymostatin and Antipain at 50–150 µg/ml (Sigma-Aldrich, UK).

### Analysis of mucus network properties

Caecal tissue was cut longitudinally, washed with PBS and kept hydrated with PBS in a 6 well plate. ESPs were applied topically at 50 µg/ml concentration for 2 h prior to analysis. 0.1 µm blue fluorescently labelled polymer microspheres (Dukes Scientific, UK) were placed on top of the luminal surface of the caecum (set as a reference) and their position analysed using a Nikon C1 Upright confocal microscope [4]. Five measurements were taken per caecum. 3D optical stacks were taken every 5 µm and combined to obtain a Z-stack at the time points stated. All images were analysed using the EZ-C1 freeviewer software (*version 3.9*).

### Agarose gel electrophoresis

Isolated mucus samples were solubilised in 6 M Urea and subsequently reduced using 50 mM dithiothreitol (DTT) and carboxylmethylated using 125 mM iodoacetamide prior to electrophoresis on a 1% (w/v) agarose gel. Whole mucus samples were separated by 0.7% agarose gel electrophoresis for 22–24 h before reduction in SSC (0.6 M NaCl, 60 mM sodium citrate) containing 0.1 M DTT for 30 min. The fractions were taken from the top of the tubes, analysed by slot blotting or agarose gel electrophoresis followed by western blotting on to a nitrocellulose membrane [15]. Mucins were detected by PAS staining or mucin-specific antisera. Staining intensity was measured using a GS-800 calibrated densitometer (Bio-Rad Laboratories, U.K).

### Rate zonal centrifugation

6–8 M guanidinium chloride (GuCl) gradients were formed in centrifuge tubes using an MSE gradient maker connected to a Gilson Minipuls 2 peristaltic pump. Mucin samples (in 4 M GuCl) were loaded onto the tops of the gradients and centrifuged in a Beckman Optima L-90K Ultracentrifuge (Beckman SW40 rotor) at 40,000RPM for 2.75 h for mucus and 3.50 h for purified mucins at 15°C. The refractive index of each fraction was measured using a refractometer; all gradients were comparable (data not shown). Data from 3–5 mice is presented as the percentage of the area under the curve (AUC) of fractions (Fr) 1–8, 9–14 and 15–18, with and without treatment.

### Purification of mucins

Mucins were purified using isopycnic density gradient centrifugation as previously described [16]. In brief, solubilised mucus was purified using caesium chloride (CsCl)/4M GuCl density gradient at a starting density of 1.4 g/ml, centrifuged in a Beckman Optima L-90K Ultracentrifuge (Beckman Ti70 rotor) at 40,000RPM for 65 h at 15°C. Periodic Acid Schiff's (PAS) rich fractions were pooled, dialysed into 0.2 M GuCl before being subjected to a second CsCl density gradient centrifugation (at a starting density of 1.5 g/ml) [16].

### Anion-exchange chromatography

The PAS-rich fractions pooled after the second CsCl density gradient was subjected to anion exchange chromatography as described previously using a Resource Q column [17]. Samples were eluted with the starting buffer (20 mM Tris-HCl at pH8) for 15 min (0.5 ml/min), followed by a linear gradient (60 min) up to 0.4 M Lithium perchlorate-10 mM piperazine at pH5 in 6 M Urea containing 0.02% 3-[(chloamidopropyl) dimethylammonio]-1-propanosulphonate [17].

### Mass spectrometry

The secreted intestinal mucus barrier was isolated using the method previously described [5]. Samples were digested with trypsin and analysed using a Q-Tof micro mass spectrometer. MS-MS data were subsequently analysed using the Uni-Prot/Swiss-Prot databases [18].

### RT-PCR

RNA was isolated from caecal epithelial cells as previously described. cDNA was generated using an IMPROM-RT kit (Promega) and SYBR Green PCR MasterMix (ABgene) was used for quantitative PCR using the ABI HT7900 PCR machine. Primer efficiencies were determined using cDNA dilutions and genes of interest were normalised against the housekeeping gene, β-actin, and expressed as a fold difference to uninfected naïve message levels. The following primer sequences were used to determine the levels of *Serpinb6a* (TGGACAAGATGGACGAAGAAGA, CCAACTTGTAGAGGGCATCGTT); *Serpina3k* (GAGCAAAGGGCAAGACCA, CAGCCACATCCAGCACAG) and *Serpinb1a* (TTCGCCTTGGAGCTGTTC, ATGCCTCCTGTAGCAGCG).

### Statistical analysis

Results are expressed as the mean ± SEM. Statistical analysis was performed using Prism v 3.2 (GraphPad Software). The statistical significance of different groups was assessed by using non-parametric tests. P<0.05 was considered to be significant.

## Results

### *T. muris* secretes products that can degrade the mucus gel

**Figure 1:**
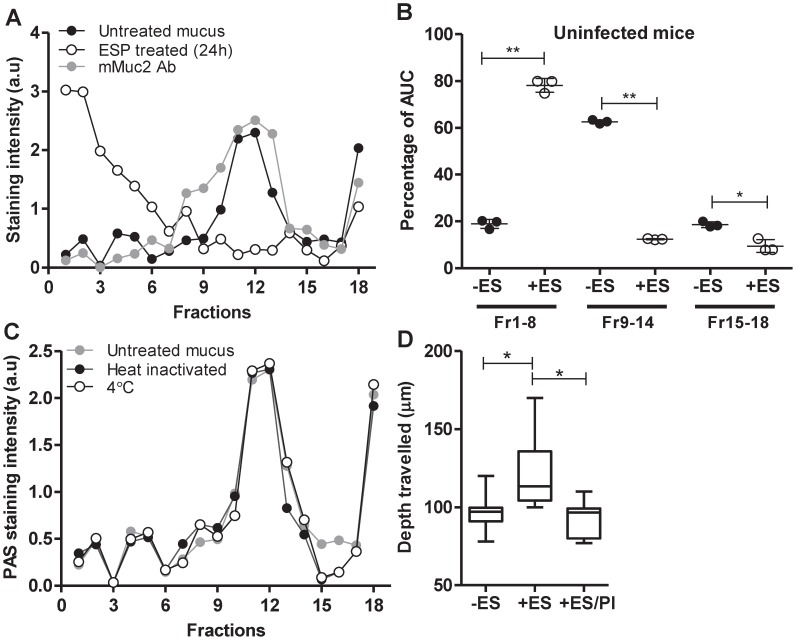
*T. muris* secretes proteases that degrade the mucus gel. (A) Mucus isolated from uninfected BALB/c mice was untreated or treated with 50 µg/ml of ESPs, and (C) heat inactivated 50 µg/ml of ESPs at 37°C or treated at 4°C for 24 h before being subjected to 6–8 M GuCl rate zonal centrifugation. Subsequently tubes were emptied by taking fractions from the top of the gradient (low density, smaller molecules) and analysed by PAS staining and or probed with the mMuc2 antibody; data presented as staining intensity arbitrary units (a.u). (B) Data presented as a percentage of area under the curve (AUC) of fractions (Fr) 1–8, 9–14 and 15–18 from untreated (−ES) and ESP-treated (+ES) mucus isolated from 3–5 mice. (D) Permeability of ESP treated (± protease inhibitors (PI)) and untreated mucus barrier was measured in caeca isolated from uninfected BALB/c mice using fluorescently labelled beads. Results represent the mean of 3–5 mice ± SD * = P<0.05; ** = P<0.01.

ESPs isolated from *T. muris* are a complex mixture of components (Figure S2A) that have been shown to contain a variety of enzymes [7]. We sought to determine whether the ESPs released from the *T. muris* nematode have a direct effect on the mucus barrier and its mucin components that are essential for the expulsion of this nematode from the host. To this end, intestinal mucus isolated from uninfected mice was incubated with ESPs for 24 h at 37°C, subsequently, treated and untreated (control) mucus samples were subjected to rate zonal centrifugation. This analysis revealed a significant change in sedimentation behaviour of the mucins after exposure to ESPs and indicated a degradative effect on the mucins that resulted in a reduction in their size; demonstrated by their lower sedimentation rate (Figure 1A–B).

The PAS staining (carbohydrate assay) profile was comparable to the mMuc2 antibody reactivity as expected since Muc2 has been shown to be the predominant intestinal mucin [19]. In addition to the major PAS and mMuc2 staining peak (fractions 9–14), a more minor PAS and mMuc2 staining species had sedimented to the bottom of the gradient (fractions 15–18). These high density mucins most likely represent the ‘insoluble’ Muc2 component of the mucus gel, as characterised previously by Carlstedt *et al.* [20]. ESPs did not have a degradative effect on Muc2 if heat inactivated prior to incubation or if the incubation was performed at 4°C (Figure 1C), suggesting that enzymes within the ESPs, most likely with proteolytic activity, were responsible for the degradation of mucin polymers. Additionally, the degradative effect was dose-dependent; with a significantly slower sedimentation rate observed with increasing concentration of ESPs (Figure S2B–C).

Mucins present within the mucus barrier are large disulphide bond-mediated polymers that determine the rheological properties of the mucus layer. When polymeric mucins are reduced to monomers, the mucus layer loses its viscous properties, thus highlighting the importance of mucin polymeric structure for the barrier properties of mucus. The significantly slower sedimentation rate of mucins after ESP treatment indicated that the mucins had a decreased size (Figure 1B), which would be predicted to alter the network properties of the mucus barrier and decrease mucus viscosity. To assess alterations in the mucin network, we measured the movement of fluorescently labelled beads within the mucus barrier. This demonstrated that the diffusion rate of the beads was significantly higher when the mucus layer was treated with ESPs (Figure 1D). Furthermore, this alteration in the mucin network was prevented when the mucus layer was concomitantly treated with ESP and protease inhibitors (PI). Collectively, these experiments suggested that ESPs were capable of degrading Muc2, resulting in a more porous mucus network.

### Mucins present in the mucus barrier during worm expulsion are less prone to degradation

**Figure 2:**
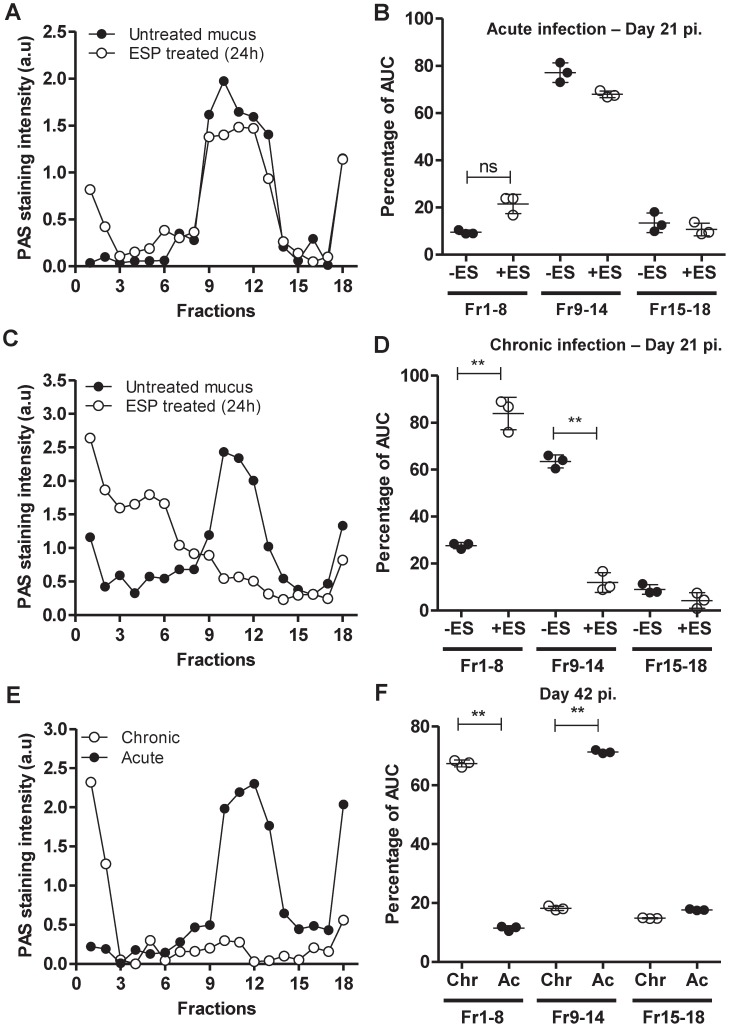
Mucins are less prone to degradation during acute infection. Mucus isolated from mice on day 21 of acute (A, B) and chronic (C, D) infection was treated with 50 µg/ml of ESPs for 24 h and subjected to 6–8 M GuCl rate zonal centrifugation. (E, F) Mucus isolated on day 42 of acute (Ac) and chronic (Chr) infection was analysed by rate zonal centrifugation. Subsequently tubes were emptied by taking fractions from the top of the gradient (low density, smaller molecules) and analysed by PAS staining; data presented as staining intensity (a.u). Data are also presented as a percentage of AUC of Fr1–8, 9–14 and 15–18 from untreated (−ES) and ESP-treated (+ES) mucus. Results represent the mean of 3–5 mice ± SD ** = P<0.01.

Previous studies from our laboratory have shown that the mucin composition of the mucus barrier is altered during *T. muris* worm expulsion (day 21 pi). Specifically, the mucus barrier is composed of Muc5ac in addition to Muc2 [3]; there are increased amounts of Muc2; and the mucins are differentially glycosylated [5]. Overall, this results in a less porous network [4]. Therefore, we sought to determine whether these changes in the mucus barrier during worm rejection affected the ability of the ESPs to degrade the mucins. To this end, mucus isolated during the course of acute (high dose infection in BALB/c mice) and chronic (low dose infection in BALB/c mice) infection, was treated with ESPs (Figure 2A–D). Untreated and ESP-treated mucus was subjected to rate zonal centrifugation prior to fractionation and analysis.

ESP treatment altered the sedimentation profiles of mucins from mice with chronic and acute infection on day 14 pi. (Figure S3A–D), as previously observed with ESP-treated mucus from uninfected naïve mice (Figure 1A–B). However, ESPs were unable to depolymerise/degrade the mucins present within the mucus isolated from mice with acute infection on day 21 pi. (Figure 2A–B) This correlates with the changes mentioned above that occur within the mucus barrier during the worm expulsion process. Interestingly, the sedimentation profile of mucins in mucus on day 21 from mice with acute infection remained unaltered even when treated with up to 300 µg/ml concentrations of *T. muris* ESPs (data not shown). In contrast, mucins in the isolated mucus from chronically infected mice on day 21 pi. were readily degraded by 50 µg/ml of *T. muris* ESPs (Figure 2C). It is important to note that a small proportion of the mucins present within the mucus isolated from the mice with chronic infection on day 21 appeared degraded before *in vitro* treatment (Figure 2C–D). However, more strikingly, mucins present within the mucus isolated from mice on day 42 pi. with chronic infection were almost completely degraded without ESP treatment (Figure 2E–F) as compared to those isolated from the mice with an acute infection. These data suggest that mucins present within the mucus barrier during long term chronic infection are degraded *in vivo* and, additionally, may be more prone to degradation by parasite proteases than those produced in mice able to expel the worms.

Surprisingly, mucins present within the mucus isolated on day 56 pi. of acute infection, which is 35 days after the expulsion of the nematode, did not fully degrade when treated with ESPs (Figure S3E–F). This suggested that the changes in the barrier [3], [4] that occur during the co-ordinated expulsion response are maintained for some time after expulsion.

### *T. muris* ESPs are unable to degrade Muc5ac

**Figure 3:**
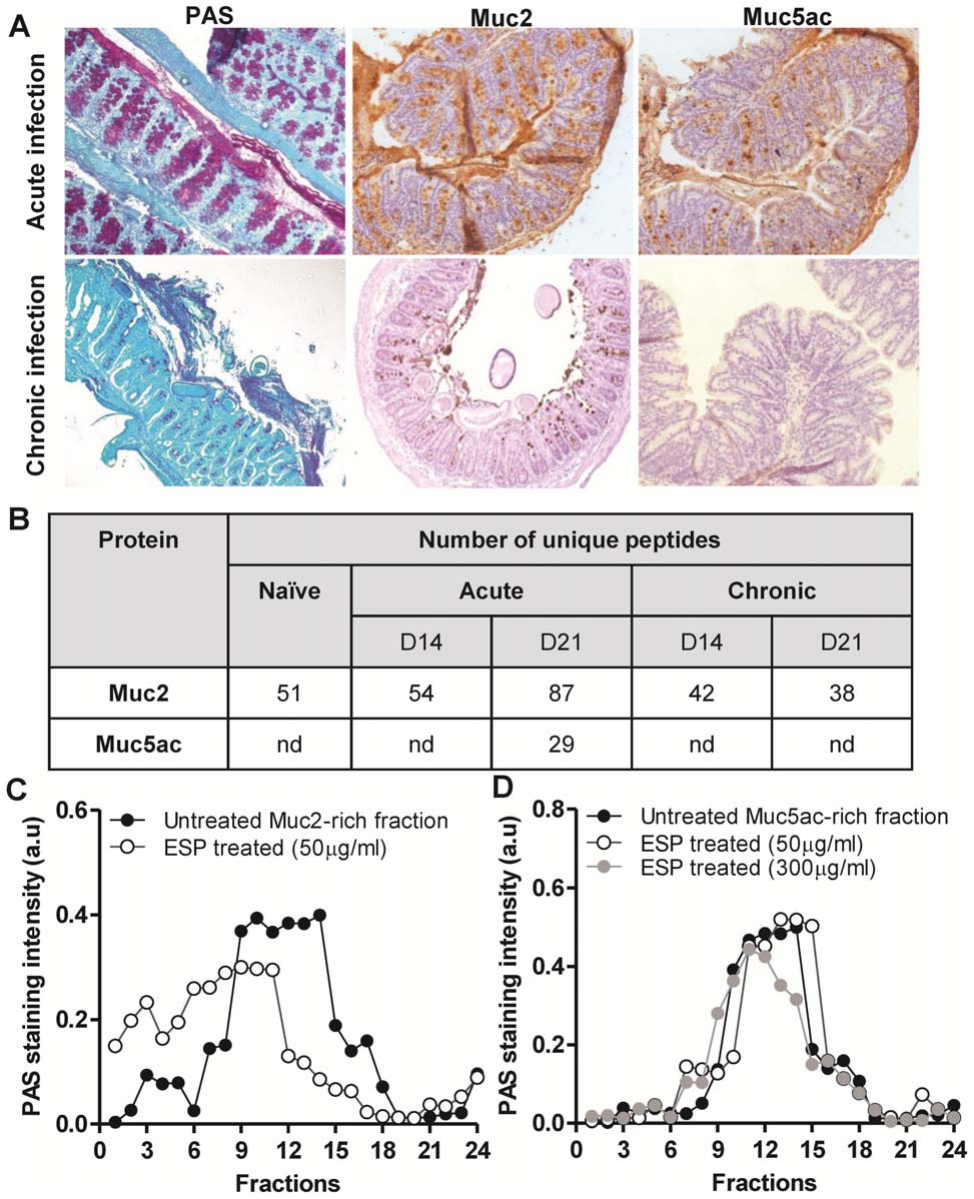
*T. muris* degrades the intestinal mucin Muc2 but not Muc5ac. (A) PAS staining and immunohistochemistry with mMuc2 and 45M1 (Muc5ac) antibodies in caecal tissue from acute and chronically infected mice on day 21 of *T. muris* infection. (B) Tabular representation of the number of unique peptides for Muc2 and Muc5ac identified by tandem mass spectrometry analysis of mucus isolated during the course of acute and chronic infection. nd = not detected. (C) Muc2-rich fraction and (D) Muc5ac-rich fraction pooled from mice with acute infection (Figure S4) were treated with ESPs (concentration as stated on the graph) for 24 h and subjected to 6–8 M GuCl rate zonal centrifugation, subsequently tubes were emptied by taking fractions from the top of the gradient (low density, smaller molecules) and analysed by PAS staining; data presented as staining intensity (a.u).

A clear difference was observed in the ESPs ability to alter the mucin component of the mucus barrier produced during worm expulsion (acute infection; day 21 pi) compared to the chronically infected mice (Figure 2). As we have previously reported, in addition to goblet cell hyperplasia and elevated levels of Muc2, IL-13 induced *de novo* expression of the Muc5ac mucin is also observed during *T. muris* expulsion [3]. Immunohistochemistry (using Muc2 and Muc5ac-specific antibodies) of caecal tissue and mass spectrometry analysis of caecal mucus confirms the increased levels of Muc2 production in acute infection and demonstrates that even during acute infection Muc2 is the predominant mucin (Figure 3A & B). Therefore, to investigate the effect of the ESPs specifically on mucins in more detail, mucins were purified from the mucus isolated on day 21 pi. of mice with acute infection. In brief, isolated mucus (pooled from 5 mice) was reduced and carboxymethylated and, the resultant mucin monomers were purified from the mucus by two isopycnic density gradient centrifugation steps. Firstly centrifugation in CsCl/4M GuCl was used to separate mucins from lower buoyant density proteins (Figure S4A) [16], followed by a second centrifugation in CsCl/0.2M GuCl to separate mucins from nucleic acids (Figure S4B) and then the distinct Muc2- and Muc5ac-rich fractions were further purified using anion exchange chromatography (Figure S4C).

Fractions enriched in monomeric Muc2 and Muc5ac were pooled from the anion-exchange column (Figure S4C), treated with 50 µg/ml of ESPs for 24 h and, analysed by rate zonal centrifugation (Figure 3C–D). Fractions analysed by slot blotting revealed that ESPs degraded Muc2, since a significant amount of Muc2 was present in the fractions at the top of the gradient after ESP-treatment (Figure 3C). In contrast, the sedimentation profile of the Muc5ac-rich fraction was unaltered after treatment with ESPs with up to 300 µg/ml (Figure 3D). This implied that ESPs specifically degrade the intestinal mucin, Muc2, but are unable to degrade the Muc5ac mucin, which we have previously shown to aid the co-ordinated worm expulsion process by reducing the nematode's vitality [3].

### ESPs depolymerises the intestinal mucin, MUC2

**Figure 4:**
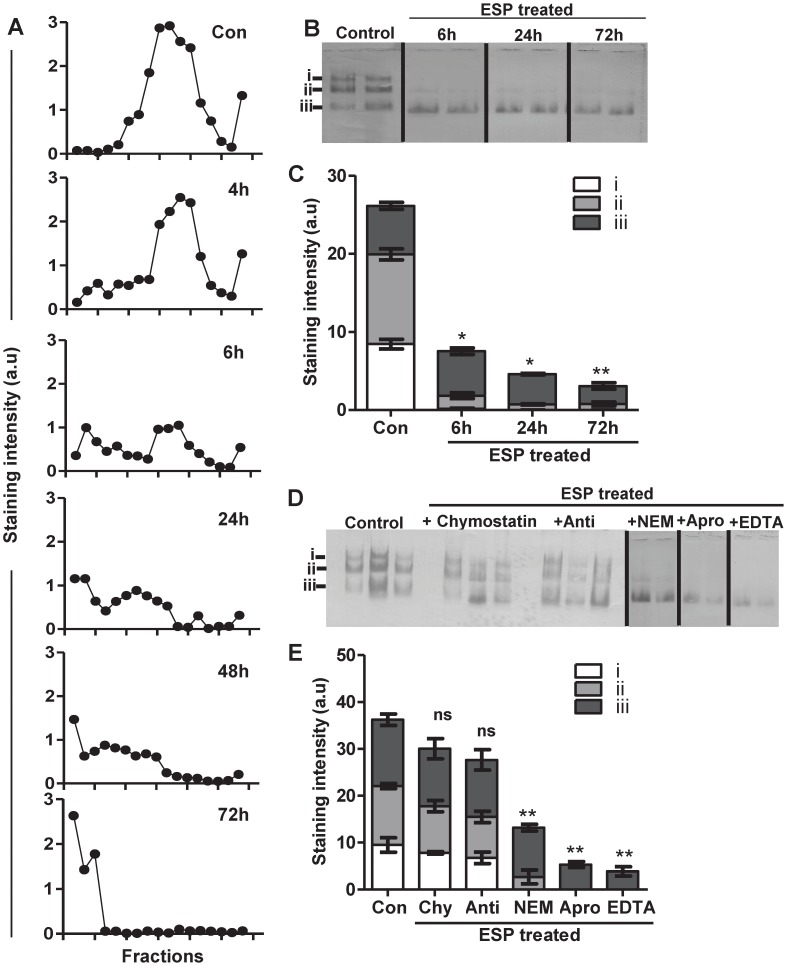
Serine proteases in *T. muris* ESPs degrade human MUC2. (A) MUC2 extracted from LS174T cell lysates was unteated (Con) or treated with 50 µg/ml of ESPs for 4, 6, 24, 48 or 72 h and subjected to rate zonal centrifugation, analysed by hMUC2 antibody staining - data represented as staining intensity (a.u). MUC2 isolated from LS174T cell lysates was treated with 50 µg/ml of ESPs in the absence (B, C) and presence (D, E) of protease inhibitors (chymostatin, antipain, NEM, Aprotinin, EDTA) for 6, 24 or 72 h, analysed by agarose gel electrophoresis, western blotted onto a nitrocellulose membrane and probed with the hMUC2 antibody. (C, E) The relative intensity of staining (a.u) combined from 3 individual experiments (mean ± SD) was measured for the bands, representing the different forms of MUC2 (i, ii, iii) ns = non-significant, * = P<0.05,** = P<0.01 compared to control.

To further explore the effect of ESPs on the MUC2 glycoprotein and in particular its degree of polymerisation, the human intestinal LS174T cell line, which synthesises and secretes mature glycosylated MUC2, was utilised as a source of MUC2. MUC2 isolated was treated with 50 µg/ml of ESPs for 4, 6, 24, 48 and 72 h were subjected to rate zonal centrifugation, which showed a time dependent shift in MUC2 distribution to the top of the gradient and a loss of staining intensity with ESP treatment (Figure 4A). Interestingly, the putative ‘insoluble’ mucin content, present at the bottom of the gradient in the control samples also gradually decreased after ESP-treatment and no ‘insoluble’ fraction was observed after 72 h of treatment (Figure 4A). Untreated and ESP treated MUC2 was subjected to agarose gel electrophoresis (Figure 4B), transferred onto a nitrocellulose membrane and probed with hMUC2 antibody. The unreduced MUC2 can be seen as at least three bands in the control samples that likely represent different multimeric forms of MUC2 (Figure 4B). Importantly, after ESP treatment the intensity of the slower migrating multimeric (i, ii) MUC2 bands were decreased significantly (Figure 4B–C). After 72 h of ESP-treatment the intensity of the fastest migrating MUC2 band (iii) decreased further (Figure 4C). An overall loss of MUC2 antibody staining was also noted, suggesting ESPs initially affect the polymerisation of MUC2 and over time degrade/cleave the MUC2 protein core.

### *Trichuris* ESPs have serine protease activity that degrades the mucin network

Proteolytic activity of ESPs was probably responsible for degrading/depolymerising the mucin network as it was demonstrated to be temperature dependent (Figure 1C) and blocked by protease inhibitors (Figure 1D). ESPs secreted by the *T. muris* nematode contain several different proteases including cysteine, serine and metalloproteases [21]. Therefore, to determine which specific protease(s) were responsible for degrading and altering the polymerisation of MUC2, ESPs were incubated in the presence of specific protease inhibitors (Table S1), prior to the treatment of MUC2. Note that the control samples contained a mixture of all the stated protease inhibitors. MUC2 was significantly degraded despite the presence of aprotinin, ethylenediaminetetraacetic acid (EDTA) and N-ethylmaleimidide (NEM) (Figure 4D–E), which inhibit chymotrypsin/trypsin, metallo- and cysteine proteases, respectively. However, it was noted that NEM, may have a partial effect on the ESP enzymatic activity as the fastest migrating band of MUC2 (iii) was intact and a low reactivity with the slower migrating MUC2 band (ii) was also observed (Figure 4D–E). More strikingly, after treatment with the serine protease inhibitors chymostatin and antipain, the ESPs were unable to alter the abundance of all forms of MUC2 (Figure 4D–E). There was a slight difference in the electrophorectic migration of the MUC2 bands noted after ESPs treatment that could not be prevented with protease inhibitor treatment, suggestive of a potential role of ESP in de-glycosylating mucins or activity of another protease in the ESPs. Therefore, overall, these data demonstrate that the depolymerisation activity of ESPs is due to serine proteases.

### ESPs cleave the polymerising N-terminus of Muc2

**Figure 5:**
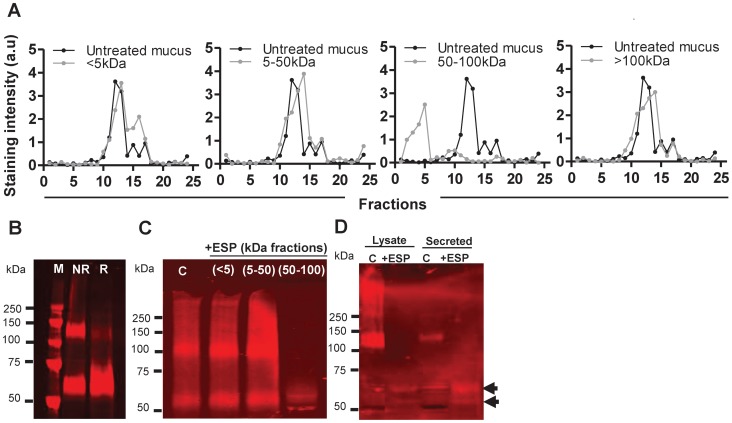
Serine proteases act on the polymerising N-terminus of MUC2. (A) MUC2 isolated from LS174T cells treated with fractionated ESPs (<5 kDa, 5–50 kDa, 50–100 kDa and >100 kDa) for 24 h at 37°C at 50 µg/ml final concentration was subjected to 6–8 M GuCl rate zonal centrifugation, fractionated and analysed by blotting onto a nitrocellulose membrane and antibody detection; data presented as staining intensity (a.u). (B) PAGE/Western blot analysis of rMuc2-D3 (detected using anti-FLAG antibody) from MKN45 cell lysates, under reduced (R) and non-reduced conditions (NR). M = molecular weight marker. (C) rMuc2-D3 was treated with <5 kDa, 5–50 kDa, 50–100 kDa fractionated ESPs for 72 h at 37°C prior to analysis by PAGE/western blot (anti-FLAG antibody). (D) rMuc2-D3 obtained from MKN45 cell lysates (L) and secretions (S) was treated with the 50–100 kDa ESP fraction for 24 h at 37°C at a final concentration of 50 µg/ml was analysed by PAGE/Western blot (detected using anti-Myc antibody). All control samples were incubated at 37°C for 72 h.

The serine protease with the enzymatic activity against mucins was fractionated from the ESPs by performing size fractionation into the following fractions <5 kDa, 5–50 kDa, 50–100 kDa and >100 kDa. MUC2 was treated with the ESP-fractions and analysed using rate zonal centrifugation. This demonstrated that 50–100 kDa ESP-fraction had activity against MUC2; whereas, no other ESP-fraction affected MUC2 (Figure 5A).

To ascertain whether the active 50–100 kDa fraction cleaves and depolymerises murine Muc2, the N-terminal D3 polymerisation domain (rMuc2-D3) of Muc2 was expressed in human MKN45 gastric cells which express mucin-specific chaperones and no endogenous MUC2; rMuc2-D3 is present intracellularly as a monomer and dimer and secreted mainly in its dimeric/multimeric forms [13]. The active 50–100 kDa ESP-fraction depolymerised the dimeric form of rMuc2-D3 protein (Figure 5C), similar to that observed when rMuc3-D3 was analysed after treatment with a reducing agent (Figure 5B). rMuc2-D3 isolated from cell lysates and secretions in conditioned media was then treated with the active ESP-fraction (Figure 5D) and analysed by SDS-PAGE/Western blot (detected using the anti-Myc antibody). In addition to the multimeric forms of rMuc2-D3 being reduced to monomers, smaller cleavage products were also observed (highlighted with arrows; Figure 5D), confirming that ESPs not only depolymerised the murine Muc2 mucin but also degraded/cleaved it into smaller fragments. Importantly, the other ESP-fractions did not have this effect on rMuc2-D3 protein (Figure 5C), and treatment with the serine protease inhibitor antipain inhibited this activity (data not shown), confirming that serine protease(s) present in the 50–100 kDa fraction were responsible for depolymerising/degrading Muc2.

### Serpins are present within the resistant mucus barrier

**Figure 6:**
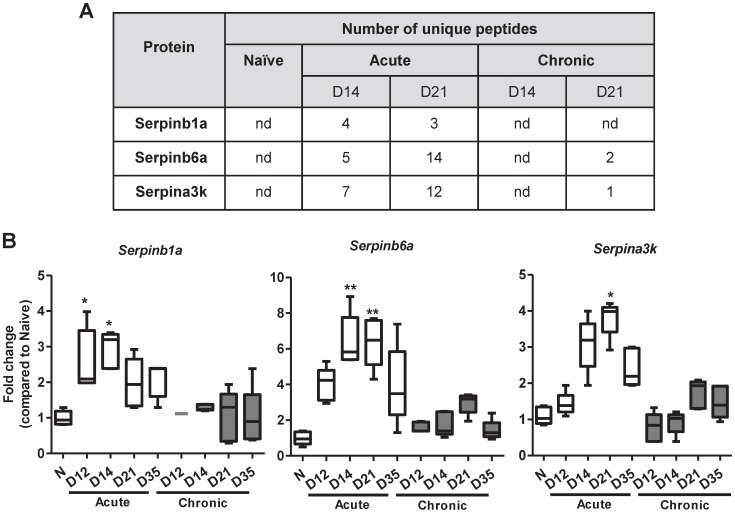
Serpins are up-regulated in the mucus barrier during worm expulsion. Tabular representation (A) of the number of unique peptides for the serine protease inhibitors (Serpins) identified by tandem mass spectrometry analysis of mucus isolated during the course of acute and chronic infection (Days 12, 14 21 and 35). nd = not detected. (B) RT-PCR was used to determine the levels of Serpinb1a, Serpinb6a and Serpina3k during the course of acute and chronic infection. Results represent the mean value of 5 mice per group compared to naïve (N) controls. Box plots show median, quartiles, and range. * = P<0.05,** = P<0.01 compared to control.

Worm expulsion is near complete by day 21 pi. [6] in the mice with the high dose infection (Figure S1), and our data clearly show that at this stage the mucins, and in particular Muc2, present within the mucus barrier were somehow protected from the degradative effects exerted by ESPs. This raised the possibility of the presence of protective non-mucin components such as protease inhibitors which may be secreted by the host within the mucus barrier to hinder the ESP activity. To this end, we isolated the secreted mucus from mice during the course of acute and chronic infection using a method previously described [5]. The proteins present within mucus were identified using trypsin digestion followed by tandem mass spectrometry (MS) analysis. Tandem MS analysis identified three members of the serine protease inhibitor family (Serpins), Serpinb6a, Serpina3k and Serpinb1a, to be present in the mucus barrier during infection. Interestingly, Serpins were not detected in the uninfected and chronically infected mice on day 14 pi., with 1–2 unique peptides identified on day 21 pi. of chronic infection (Figure 6A, B). In contrast, the mucus barrier isolated during acute infection, contained higher levels of serpins on day 14 and day 21 pi. RT-PCR analysis demonstrated that mRNA levels encoding *Serpinb1a*, *Serpinb6a* and *Serpina3k* (Figure 6B), identified in the mucus barrier with MS analysis, were elevated in the caecal epithelium of mice during the T_H_2-mediated immune response; however no major changes were observed during chronic infection. The host therefore potentially up-regulates serine protease inhibitors during the worm expulsion process in order to prevent mucin degradation *in vivo*, and subsequent inflammation and aid the rejection of the nematode from the intestine.

## Discussion

Mucins are an essential part of the T_H_2-mediated immune response that enforces intestinal expulsion of the nematode, *Trichuris muris* [1], [3], [4]. In this study, we demonstrate how the *Trichuris* nematode has evolved ways to promote its survival within its intestinal niche by degrading mucins; the molecular framework of the host protective mucus barrier. This is the first study to describe that serine protease(s) released by the nematode can cleave the polymerising domain of Muc2, the major intestinal mucin, leading to a more porous mucus barrier network. Here, we clearly demonstrate that the Muc2 mucin, but not Muc5ac, was degraded by secretions from *T. muris* nematodes; and serine protease inhibitors are present in the mucus barrier during worm expulsion, which may hinder the degradative effects of ESPs. The degradation of Muc2, but not Muc5ac, by parasite proteases provides a plausible explanation for our previous observation that, whilst Muc2 aids worm expulsion [4] only Muc5ac is essential for expulsion [3]. Therefore, components of the ESPs are produced by the nematode to alter the properties of the mucus barrier and thus facilitate its own survival and/or improve the conditions within its niche.

The *Trichuris* nematodes are extremely successful within the host, because they not only have the ability to survive an immune attack but are thought to actively subvert the immune responses generated by the host by exerting immunomodulatory effects [6]. These nematodes have been shown to produce complex secretions containing proteases and proteins that have immunogenic properties, thus described as ‘excretory secretory antigen’, but which are likely to have a protective function [7], in particular in maintaining infection within its mucosal niche. Mucins are responsible for the physical properties of the mucus barrier. These large heavily glycosylated molecules polymerise, mediated by disulphide bond formation involving the cysteine-rich domains at the N- and C-termini of the mucin polypeptide, which gives the barrier its viscous properties [22]. The parasite proteases have evolved the ability to act on the polymerising D3 domain of the intestinal mucin, Muc2, which is thought to be largely protease resistant [23], due to the high level of intra-molecular disulphide bonding. The dimeric form of a recombinant mucin protein was depolymerised into monomers after ESP-treatment and this was prevented if serine proteases inhibitors were used and a smaller fragment than the monomeric form was also observed. Clearly, the depolymerisation/degradation of mucins impacts on the properties of the mucus barrier, as the barrier was more porous after ESP treatment, which also corroborates our previous finding of a more porous mucin network in mice with chronic infection [4].

The host has developed ways to counteract the depolymerising ability of ESPs. We observed a significant difference in the ESPs ability to degrade mucins isolated during acute and chronic infection. As described previously, under the T_H_2-mediated immune response the mucin component of the mucus barrier changes during worm expulsion with a prominent increase in Muc2 [4], *de novo* expression of the mucin Muc5ac [3] and change in glycosylation observed [5]. ESPs were unable to degrade the mucins in mucus isolations on day 21 of acute infection, which is the peak of the immune response [6]. Interestingly, when the mucins were purified we could show that the ESPs have the ability to degrade mouse and human Muc2/MUC2, but not the Muc5ac mucin, which is not usually expressed in the intestinal epithelium. It is plausible that these nematodes have evolved a capability to degrade the major mucin in the intestine, Muc2, whilst being unable to degrade Muc5ac. This suggests that the *Trichuris* proteases are specifically acting on a peptide sequence within the MUC2/Muc2 protein core that appears conserved between mouse and human. The presence of the IL-13 induced protease-resistant Muc5ac during nematode expulsion will maintain mucus viscosity and retention of anti-helminthic factors in the mucus and subsequently be detrimental for nematode viability as previously demonstrated [3].

Interestingly, in addition to Muc5ac and increased levels of Muc2, we show that Serpins were upregulated and were present within the mucus barrier of mice resistant to chronic infection. The presence of Serpins, and possibly other protease inhibitors in the mucus barrier during worm expulsion could explain why mucins, in particular Muc2, were protected from degradation when treated with ESPs but Muc2 when purified, was susceptible to degradation. Another possibility for the lack of degradation of mucus could be due to the increased concentration of mucins and other proteins within the mucus barrier [4]. The increased levels of proteins could result in more competition for ESPs to cleave sites and, therefore, make the mucins less susceptible to degradation as illustrated previously in respiratory mucus [24]. The changes to the properties and composition of the mucus barrier could hinder ESPs activity, which could also explain the decrease in vitality of the nematode during worm expulsion [3], [4].

This is not the first time pathogen exo-products have been shown to degrade mucins, several other pathogens have adopted a similar mechanism to survive within the mucosal layer. *Helicobacter pylori* secretes ‘mucinases’ which allow its corkscrew-like motion through the mucus layer [25]. Protozoan parasites such as *Entamoeba histolytica* [26], *Trichomonas vaginalis* [27] and *Naegleria fowleri* [28] all release cysteine proteases which have the ability to degrade mucins. We demonstrated that cysteine protease inhibitors (NEM and aprotinin) only very partially limited the activity of *Trichuris* ESPs to degrade MUC2. Treatment with chymostatin and antipain inhibited the depolymerisation of MUC2 implicating trypsin and/or serine protease activity. However, since degradation was not inhibited by aprotinin (cysteine and trypsin protease inhibitor), it is most likely that serine protease activity is responsible for degrading Muc2/MUC2. ESPs contained serine protease(s) of molecular weight range 50–100 kDa with the depolymerising activity against the gel-forming mucins. Interestingly, serine proteases of molecular weight of 85 and 105 kDa have been reported to be isolated from *T. muris* ESPs previously [21]. Whilst serine proteases appeared to be the major Muc2 protease, our data imply that cysteine proteases present in the ESPs were in part responsible for the affects observed on the insoluble Muc2/MUC2-gel. Serine and cysteine proteases, therefore, may act in concert to disrupt the polymeric mucin network. Supporting, this hypothesis further is the presence of Serpins within the mucus barrier prior to and during worm expulsion, which may hinder the ability of ESPs to break down the mucus barrier.

**Figure 7:**
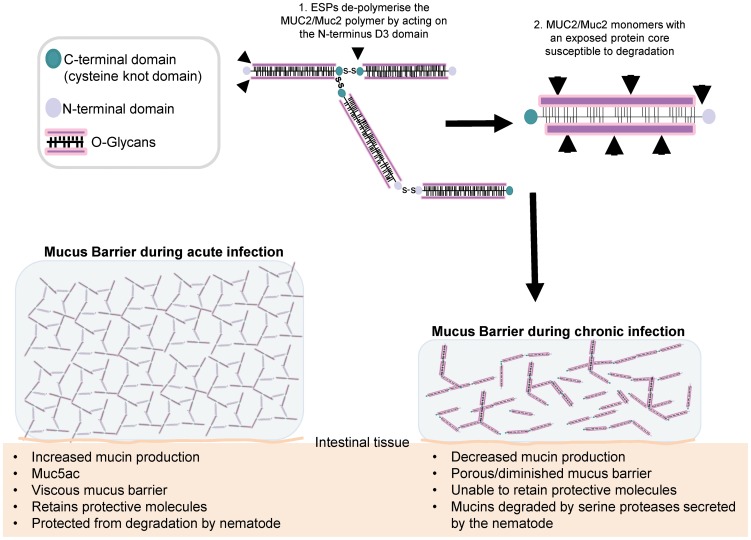
Schematic overview of the mechanism by which *T. muris* serine proteases degrade the mucus barrier. Diagram illustrating the likely sequence of events leading to a porous mucus barrier. *T. muris* ESPs cleave the Muc2 polymerising D3 domain, which leads to decreasing the size of mucin polymers, thus making the protein core susceptible to further degradation events. Overall, this results in reducing the viscosity of the mucus barrier making it more porous which could decrease the retention of anti-helminthic host factors in the mucus and therefore, may help promote the survival/establishment/persistence of the nematode within its intestinal niche.

There is an added level of unique complexity in the assembly of the intestinal MUC2: an uncharacterised ‘non-reducible linkage’ which results in an ‘insoluble’ gel enabling MUC2 to form a barrier resistant to the harsh environment of the intestine [20]. In addition, other proteins such as Fc Ig binding protein (Fcgbp) have been shown to associate with Muc2/MUC2 and could potentially act as a cross-linkers [19]. Interestingly, for the first time we demonstrate that *Trichuris* ESPs can degrade the Muc2/MUC2 polymers into smaller subunits and may have a further effect on the MUC2 protein as there was a change in the electrophorectic migration suggesting ESPs may be involved in de-glycosylating/degrading the MUC2 protein. Interestingly, as the mucins produced *in vivo* during acute infection are protected, the mucins present in the mucus barrier of susceptible mice also have reduced glycosylation [5], which may make them more prone to the effects of ESPs. Taken together, the data suggests that the serine protease activity of the ESPs cleaves the mucin polymerising domain (Figure 7) resulting in mucin monomers [29] which can be cleaved/degraded into smaller fragments (Figure 7). This will subsequently result in a mucus barrier that is more porous and, will therefore, exacerbate inflammation and aid persistence of infection.

Although the stability and turnover of the ESPs is not known *in vivo*, ESPs can clearly increase the porosity of the mucus layer by depolymerisation of mucins, which would hinder the retention of host protective factors within the mucus barrier. Furthermore, bearing in mind the niche in which *Trichuris* lives there will be a major interface between the adult parasite and secreted mucins within the mucus layer via the posterior half of the worm, which protrudes out of the epithelium into the caecal lumen. The posterior section of adult worms is involved in mating and ultimately egg deposition, and it is tempting to speculate that modification of the mucus barrier properties, perhaps via the proteolytic activity described here would and, allow optimal mobility of the posterior end of the worm, facilitating efficient mating and egg laying during chronic infection. Many questions remain unanswered including identification of the specific protease(s) and the cleavage site on the mucin, the site of protease(s) production and the details of the host anti-protease response. Answering these will deepen our understanding of the host-parasite relationship of this group of ubiquitous and important gastrointestinal dwelling nematodes.

## Supporting Information

Figure S1 **Course of acute and chronic infection.** BALB/c mice were infected with a high dose (∼150) or a low dose (<15) of *T. muris* eggs. Worm burdens were assessed on Day 12, 21 and 35 pi. to confirm establishment of infection, and acute and chronic infection. Box plots show median, quartiles, and range.
(TIF)

Figure S2 ***T. muris*** **ESPs contain a variety of proteins.** (A) 50 µg of *T. muris* ESP was analysed by SDS-PAGE and stained with coomassie blue. (B) Mucus from uninfected mice was treated with increasing concentrations of ESPs (as specified) and subjected to rate zonal centrifugation; data represented as staining intensity (a.u). (C) Data presented as a percentage of area under the curve of fractions (Fr) 1–9 and 10–18 from untreated and ESP-treated mucus isolated from 3 mice per group mice ± SD. *** = P<0.01 compared to control.
(TIF)

Figure S3 **ESPs are able to degrade the mucus gel on day 14 of acute and chronic infection.** Mucus isolated from mice with acute infection on day 14 (A, B) or day 56 (E, F) of infection or from mice with chronic infection on day 14 of infection (C, D) was untreated (−ES) or treated (+ES) with 50 µg/ml of ESPs for 24 h. Samples were subjected to 6–8 M GuCl rate zonal gradients and subsequently tubes were emptied by taking fractions from the top of the gradient and analysed by PAS-staining. Data represented as staining intensity (a.u). (B, D, F) Data presented as a percentage of AUC of Fr1–8, 9–14 and 15–18 from untreated and ESP-treated mucus isolated from 3 mice per group mice ± SD. ** = P<0.01.
(TIF)

Figure S4 **Purifying mouse Muc2 and Muc5ac from the mucus gel.** (A) CsCl-density gradient centrifugation in 4 M GuCl of mucus pooled from 4 mice with acute infection on day 21. Fractions were analysed by PAS and coomassie blue staining and, density was measured. PAS-rich fractions were pooled as shown by the corresponding dashed lines and subjected to CsCl-density gradient in 0.2 M GuCl (B). Fractions were analysed by PAS and PAS-rich fractions were pooled and subjected to anion-exchange chromatography (C). Fractions taken were blotted onto a nitrocellulose membrane and probed with mMuc2 or Muc5ac antibody. Muc2- and Muc5ac-rich fractions were analysed by agarose gel electrophoresis/western blotting and stained with mMuc2 antibody (alternate fractions 19–37) or Muc5ac antibody (alternate fractions 28–46). Data represented as staining intensity (a.u). Data representative of 2 individual experiments.
(TIF)

Table S1 **Protease inhibitors and their activity against proteases.**
(TIF)
