# Supplementary figures and images for: Serine Protease(s) Secreted by the Nematode Trichuris muris Degrade the Mucus Barrier
Source: PLoS Negl Trop Dis. 2012 Oct 11;6(10):e1856. doi: 10.1371/journal.pntd.0001856 (PMC3469553; doi:10.1371/journal.pntd.0001856)

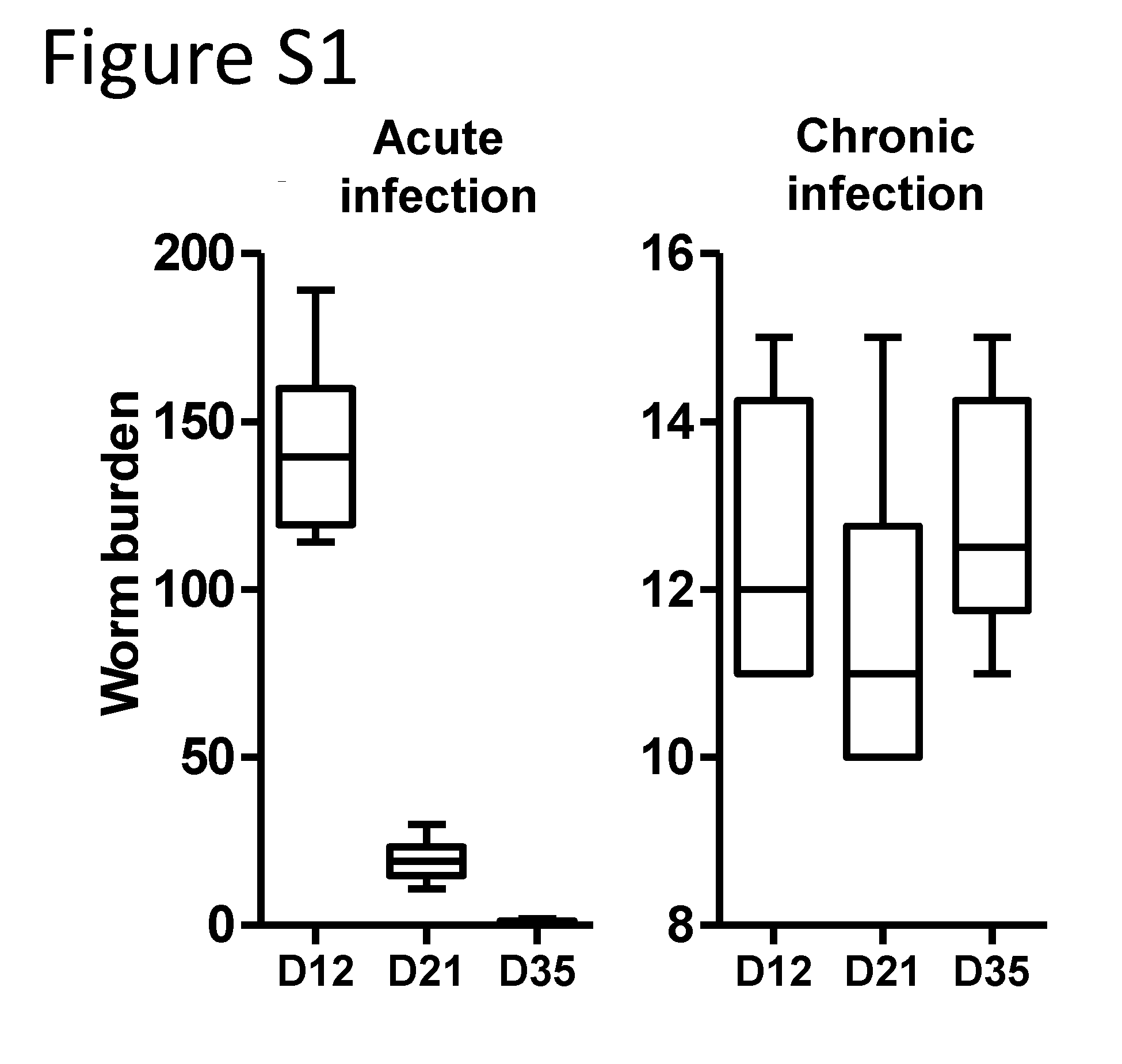

Supplement: Figure S1 — Course of acute and chronic infection. BALB/c mice were infected with a high dose (∼150) or a low dose (<15) of T. muris eggs. Worm burdens were assessed on Day 12, 21 and 35 pi. to confirm establishment of infection, and acute and chronic infection. Box plots show median, quartiles, and range. (TIF) [file pntd.0001856.s001.tif]

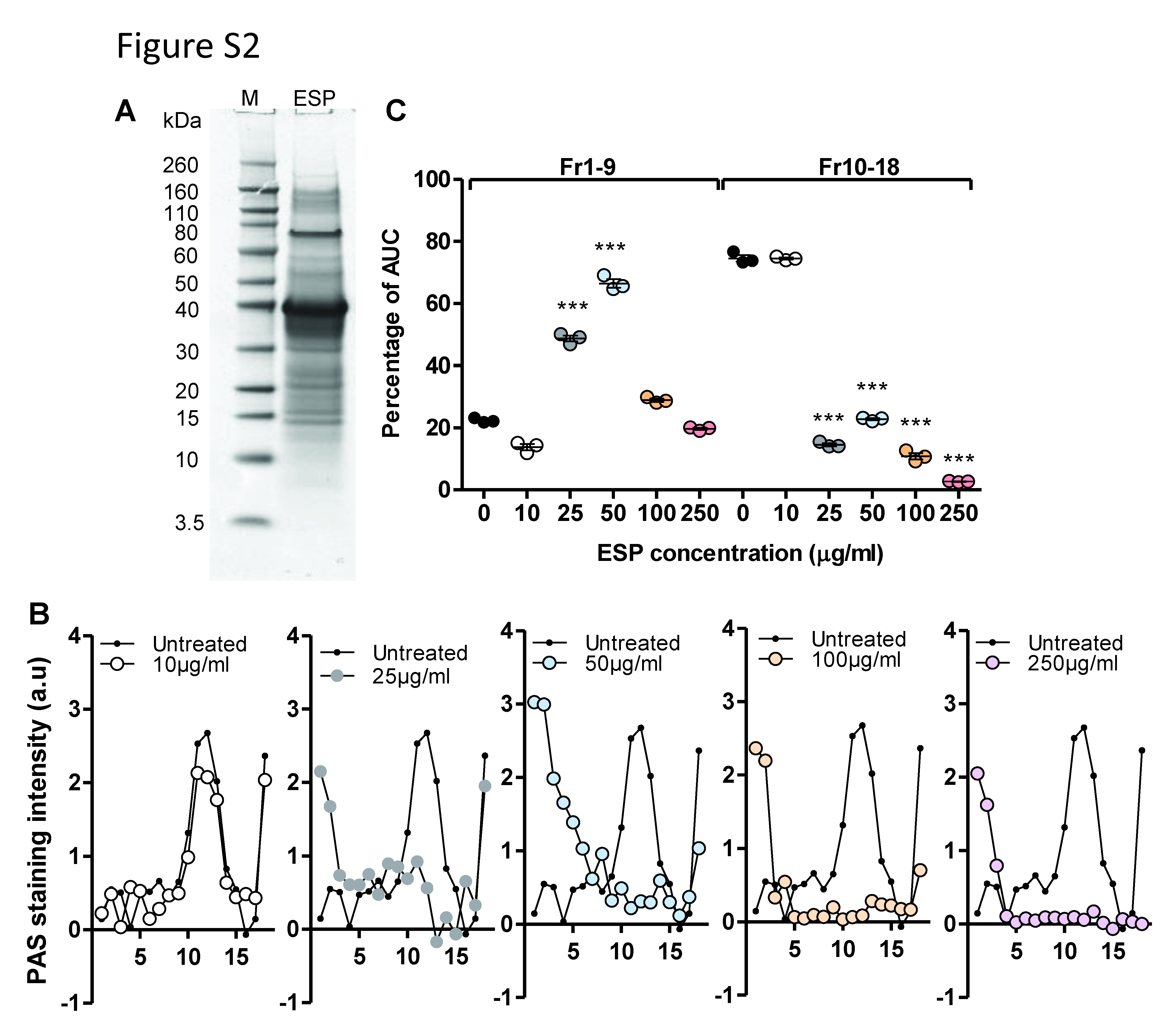

Supplement: Figure S2 — T. muris ESPs contain a variety of proteins. (A) 50 µg of T. muris ESP was analysed by SDS-PAGE and stained with coomassie blue. (B) Mucus from uninfected mice was treated with increasing concentrations of ESPs (as specified) and subjected to rate zonal centrifugation; data represented as staining intensity (a.u). (C) Data presented as a percentage of area under the curve of fractions (Fr) 1–9 and 10–18 from untreated and ESP-treated mucus isolated from 3 mice per group mice ± SD. *** = P<0.01 compared to control. (TIF) [file pntd.0001856.s002.tif]

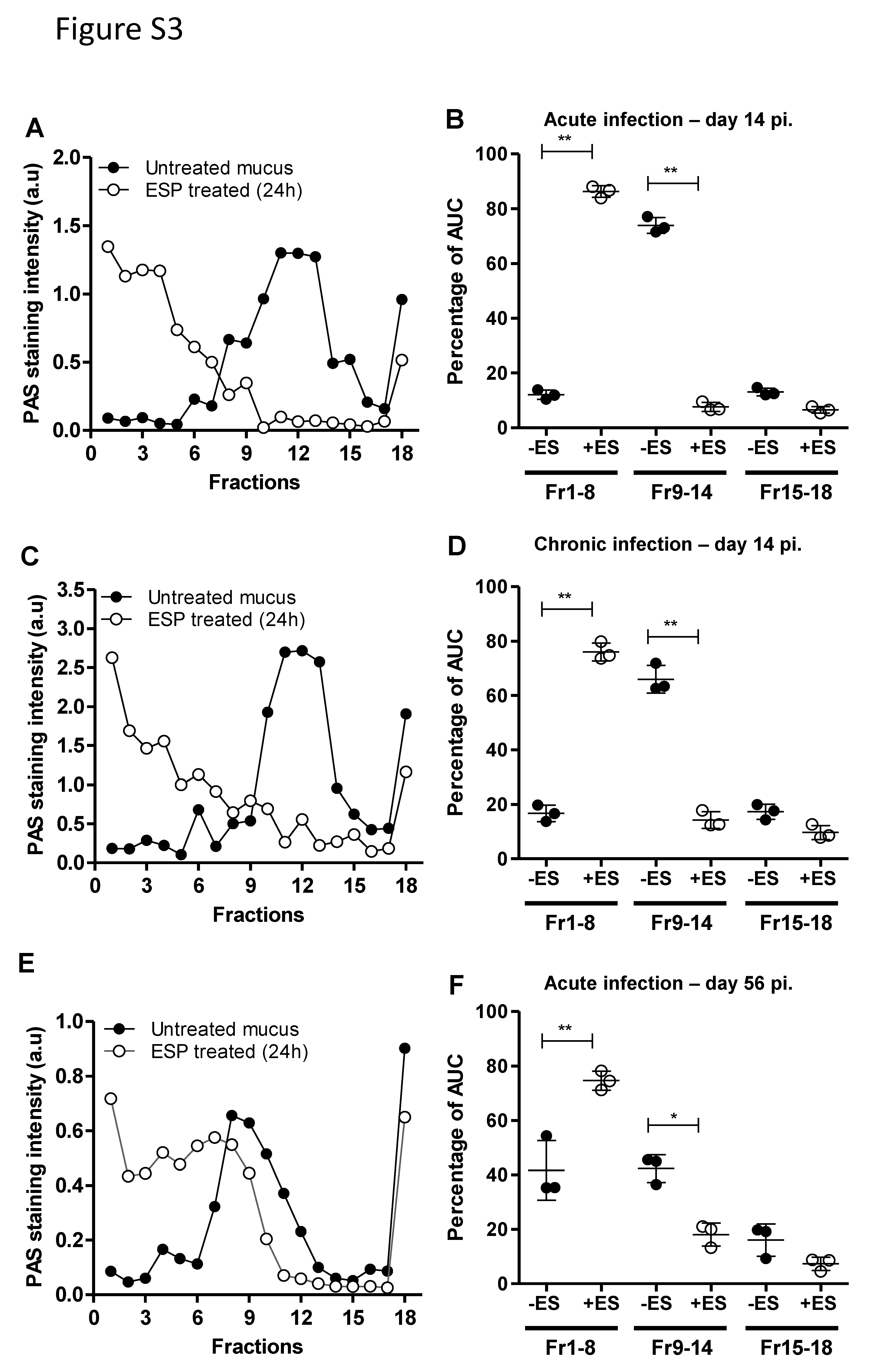

Supplement: Figure S3 — ESPs are able to degrade the mucus gel on day 14 of acute and chronic infection. Mucus isolated from mice with acute infection on day 14 (A, B) or day 56 (E, F) of infection or from mice with chronic infection on day 14 of infection (C, D) was untreated (−ES) or treated (+ES) with 50 µg/ml of ESPs for 24 h. Samples were subjected to 6–8 M GuCl rate zonal gradients and subsequently tubes were emptied by taking fractions from the top of the gradient and analysed by PAS-staining. Data represented as staining intensity (a.u). (B, D, F) Data presented as a percentage of AUC of Fr1–8, 9–14 and 15–18 from untreated and ESP-treated mucus isolated from 3 mice per group mice ± SD. ** = P<0.01. (TIF) [file pntd.0001856.s003.tif]

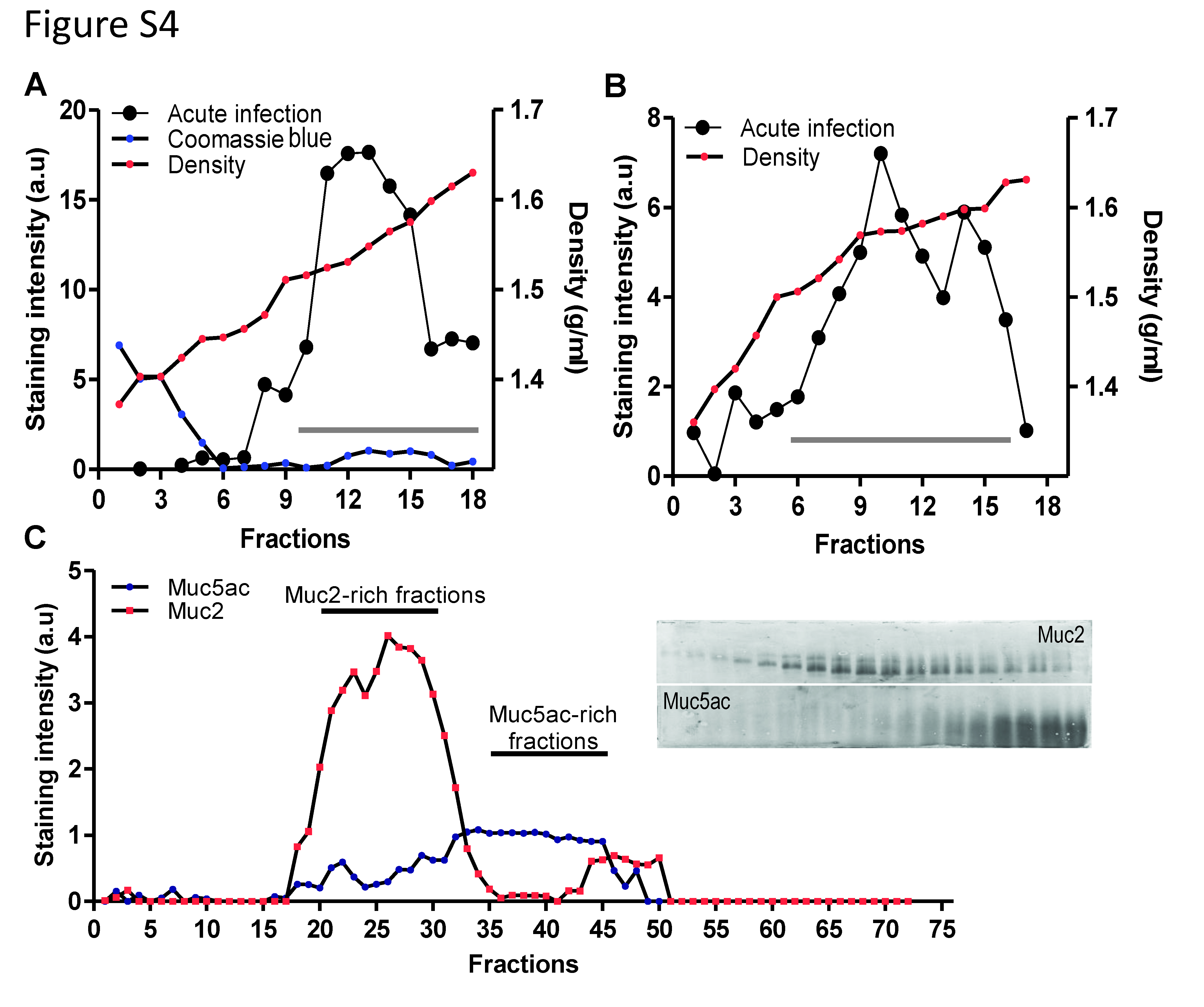

Supplement: Figure S4 — Purifying mouse Muc2 and Muc5ac from the mucus gel. (A) CsCl-density gradient centrifugation in 4 M GuCl of mucus pooled from 4 mice with acute infection on day 21. Fractions were analysed by PAS and coomassie blue staining and, density was measured. PAS-rich fractions were pooled as shown by the corresponding dashed lines and subjected to CsCl-density gradient in 0.2 M GuCl (B). Fractions were analysed by PAS and PAS-rich fractions were pooled and subjected to anion-exchange chromatography (C). Fractions taken were blotted onto a nitrocellulose membrane and probed with mMuc2 or Muc5ac antibody. Muc2- and Muc5ac-rich fractions were analysed by agarose gel electrophoresis/western blotting and stained with mMuc2 antibody (alternate fractions 19–37) or Muc5ac antibody (alternate fractions 28–46). Data represented as staining intensity (a.u). Data representative of 2 individual experiments. (TIF) [file pntd.0001856.s004.tif]

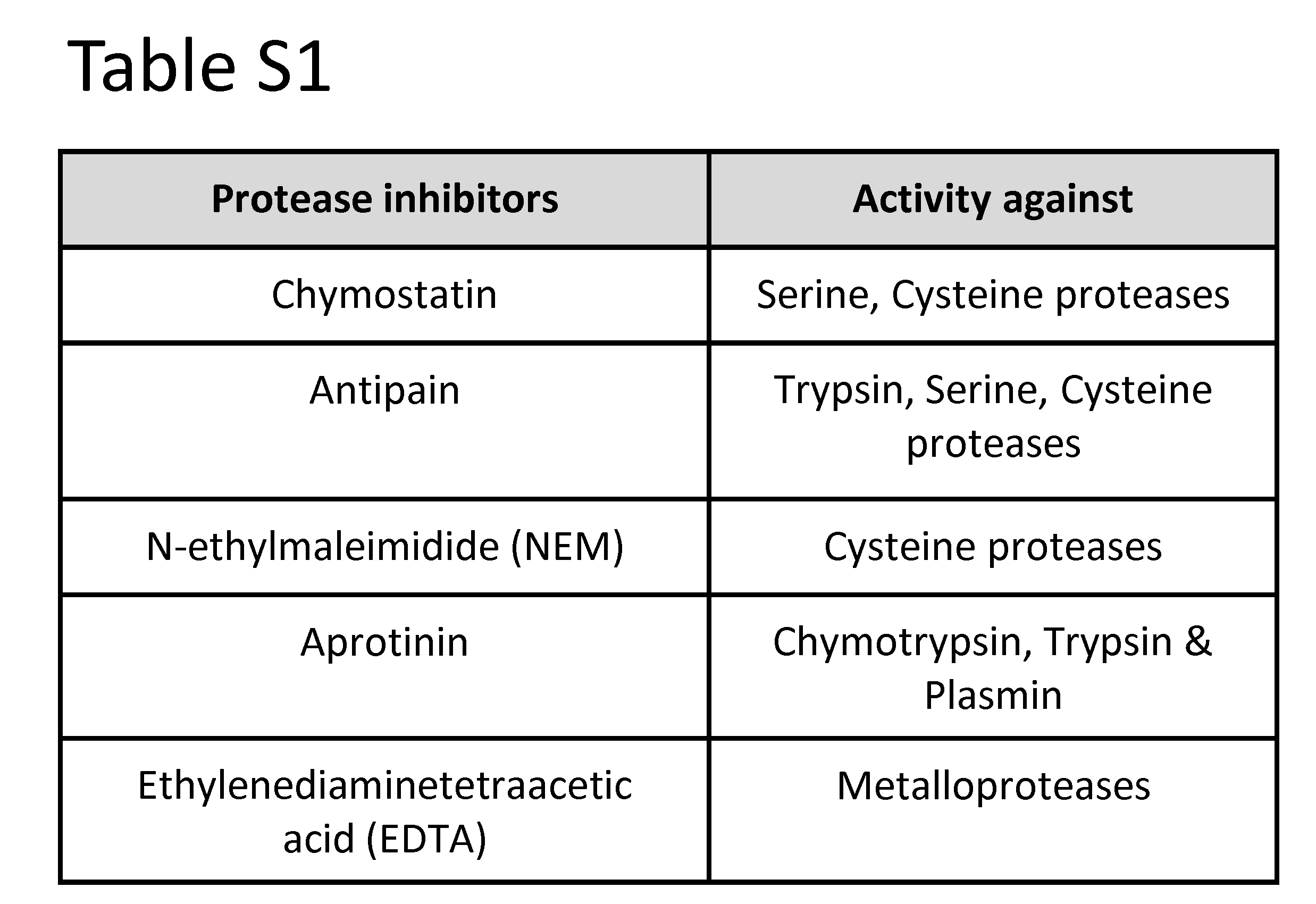

Supplement: Table S1 — Protease inhibitors and their activity against proteases. (TIF) [file pntd.0001856.s005.tif]
